# Valorization Strategies in CO_2_ Capture: A New Life for Exhausted Silica-Polyethylenimine

**DOI:** 10.3390/ijms241914415

**Published:** 2023-09-22

**Authors:** Irene Coralli, Demetra Giuri, Lorenzo Spada, Jacopo Ortolani, Laura Mazzocchetti, Claudia Tomasini, Lee A. Stevens, Colin E. Snape, Daniele Fabbri

**Affiliations:** 1 Department of Chemistry “Giacomo Ciamician”, University of Bologna, Technopole of Rimini, Via Dario Campana 71, 47922 Rimini, Italy; irene.coralli2@unibo.it (I.C.); claudia.tomasini@unibo.it (C.T.); dani.fabbri@unibo.it (D.F.); 2 Department of Industrial Chemistry “Toso Montanari”, University of Bologna, Viale Risorgimento 4, 40136 Bologna, Italy; jacopo.ortolani3@unibo.it (J.O.); laura.mazzocchetti@unibo.it (L.M.); 3 Faculty of Engineering, University of Nottingham, The Energy Technologies Building, Nottingham NG7 2TU, UK; lee.stevens@nottingham.ac.uk (L.A.S.); colin.snape@nottingham.ac.uk (C.E.S.)

**Keywords:** carbon capture, silica-PEI adsorbent, pyrolysis, solvent extraction

## Abstract

The search for alternative ways to give a second life to materials paved the way for detailed investigation into three silica-polyethylenimine (Si-PEI) materials for the purpose of CO_2_ adsorption in carbon capture and storage. A solvent extraction procedure was investigated to recover degraded PEIs and silica, and concomitantly, pyrolysis was evaluated to obtain valuable chemicals such as alkylated pyrazines. An array of thermal (TGA, Py-GC-MS), mechanical (rheology), and spectroscopical (ATR-FTIR, ^1^H-^13^C-NMR) methods were applied to PEIs extracted with methanol to determine the relevant physico-chemical features of these polymers when subjected to degradation after use in CO_2_ capture. Proxies of degradation associated with the plausible formation of urea/carbamate moieties were revealed by Py-GC-MS, NMR, and ATR-FTIR. The yield of alkylpyrazines estimated by Py-GC-MS highlighted the potential of exhausted PEIs as possibly valuable materials in other applications.

## 1. Introduction

Carbon dioxide emissions in the atmosphere from anthropogenic sources are one of the main concerns in the modern era due to the impact this greenhouse gas has on climate change [1,2,3,4,5].

The goal of reducing atmospheric CO_2_ levels has led to the implementation of actions to reverse this impact by considering different approaches to mitigate such effects including capture, utilization, and storage (CCUS) of carbon dioxide [6,7,8,9,10].

Several technological processes for CO_2_ removal have been investigated, such as membrane separation, physical and chemical absorption with solvents, and physical and chemical adsorption on solid materials [6]. Despite being a mature technology, the absorption of CO_2_ in liquid phase amines suffers from high regeneration costs, oxidative degradation, and equipment corrosion. For this reason, interest has shifted towards the search for a recyclable solid sorbent with competitive performance. Among these solid materials, zeolites and zeotypes [11,12,13,14,15], carbonaceous substances [12,15,16], and metal-organic frameworks (MOFs) [12,15,17,18] are typical examples of physical CO_2_ adsorbents, while amine-supported materials have been exploited as chemical adsorbents [12,15]. Some of the disadvantages of most physical sorbents are the large temperature and/or pressure gradients between the adsorption/desorption steps, the low selectivity towards CO_2_, as well as the poor performance at high temperatures and low tolerance in the presence of water vapor [19]. Chemical adsorbents, in contrast, are obtained by the modification of the surface chemistry of porous substrates by functionalization with amine groups [20,21,22,23,24,25]. When properly designed, these materials exhibit fast CO_2_ adsorption and desorption, high adsorption capacity and low energetic demand for the regeneration step. The chemical adsorption process involves the formation of reversible bonds between the CO_2_ and the amines. The reaction between polyethylenimines (PEIs) and CO_2_ has been proposed to proceed by the formation of a carbamate ion or carbamic acid [26,27]. Drage et al., 2008, reported that under dry gas conditions and high temperatures (above 135 °C) the formation of urea-type linkages can occur, deactivating the impregnated PEI. However, this problem can be overcome in the presence of moisture. In fact, as many groups reported, under proper humidity conditions, bicarbonate is formed instead of carbamates, enhancing the CO_2_ uptake [19,28,29].

Whilst a great effort is devoted to the development of increasingly efficient carbon capture systems [10,17,30,31,32], parallel routes aim at extending the lifetimes of materials, minimizing waste generation. In this context, different technologies using pyrolysis have been involved in the valorization of solid wastes, such as biomass or plastics [33,34,35,36].

In the present paper, the silica-polyethyleneimine (Si-PEI) system has been investigated as a sorbent technology. Both fresh (used as a reference material) and spent (after CO_2_ adsorption) Si-PEI samples were investigated to explore the regeneration capability of the spent materials (aiming to recover the mesoporous silica support) and the valorization strategies for waste (to obtain valuable compounds of industrial interest). Methods involving pyrolysis, chemical extraction, and their combination were developed and the materials underwent a step-by-step characterization of their spectroscopic, thermal, and mechanical properties.

**Figure 1:**
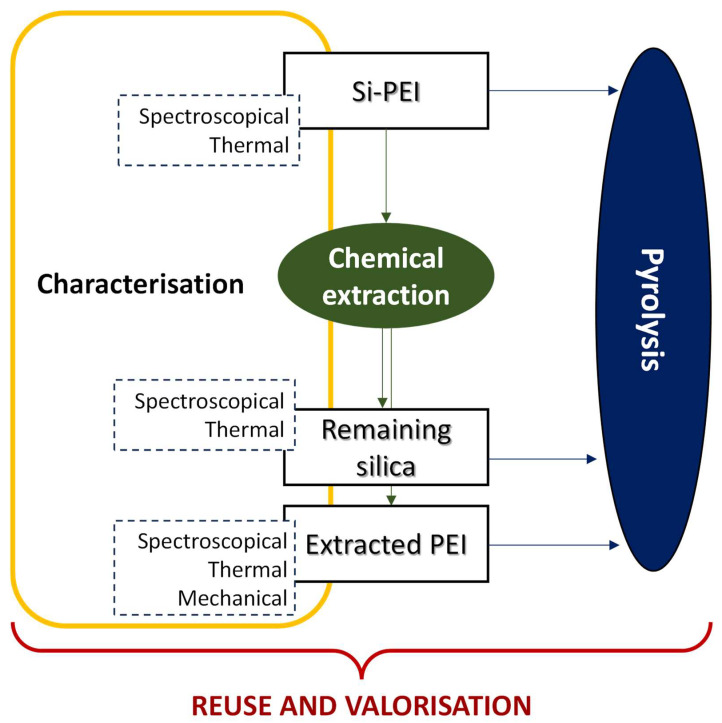
Scheme of the outline of the present investigation.

The manuscript is structured as follows (see Figure 1). Firstly, the starting materials are described along with their macroscopical properties. Next, a method for the chemical extraction of PEIs from Si-PEI samples is proposed and the characterizations of the extracted PEIs and remaining silica matrix are presented. Lastly, the valorization of the spent materials by pyrolysis is discussed, encompassing both the formation of valuable compounds and the impact of the chemical extraction on the regeneration of silica support. Concomitantly, the characterization of the sorbed and extracted PEIs was investigated with the aim of enhancing the current understanding of its degradation.

## 2. Results and Discussion

### 2.1. Si-PEI

Two spent Si-PEI sorbents (SP2 and SP3, which stand for Si-PEI with 2% and 3% of loaded CO_2_, respectively), obtained from different conditions of CO_2_ trapping were investigated. The original fresh Si-PEI (SP0) and its pure components, namely silica (PQ4) and PEI 5k, were also studied (see Section 3).

**Figure 2:**
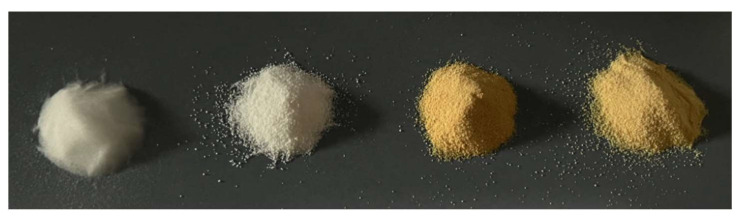
From left to right: PQ4; SP0; SP2; and SP3 samples.

Macroscopically, as shown in Figure 2, one of them is white (SP0) and two are yellow-orange colored Si-PEI powders (SP2 and SP3) containing some visible black residues. Focusing on the particle size, it is clearly visible to the naked eye that pure silica consists of smaller grains than the other ones.

Thermogravimetric analysis (TGA) curves were recorded on fresh Si-PEI (SP0) and on its respective pure components (silica PQ4 and commercial PEI 5k, see Section 3), in order to investigate their thermal stability and thermodegradative behavior. All the TGA curves were recorded in an inert atmosphere, in order to highlight the thermodegradative processes while avoiding oxidation. However, reaching 600 °C in an inert atmosphere was not sufficient to completely remove the organic fraction (char residue). It is worth pointing out that all the analyzed samples showed significant weight losses below 150 °C, which was attributed to the release of water and other low-boiling compounds.

These components were observed in TGA of commercial PEI 5k (see Supporting Information, Figure S1), whose curve clearly displays about 50% volatiles uptake. The polymer appears highly stable up to about 300 °C (T_onset_ = 304 °C) and fully decomposes even in an inert atmosphere with a subsequent double weight loss, and no significant residue persists at 600 °C in nitrogen. The plain PQ4 silica also displayed a slight volatile content; however, within the 600 °C heating ramp in N_2,_ no significant weight drop was observed, and there was a slightly decreasing trend (Figure S2A). Application of a 600 °C isotherm step (Figure S2B), with a switch from N_2_ to air atmosphere, revealed a further slight weight drop (about 2%wt), due to the oxidation of char which led to a significantly high (about 95%) final residue.

**Figure 3:**
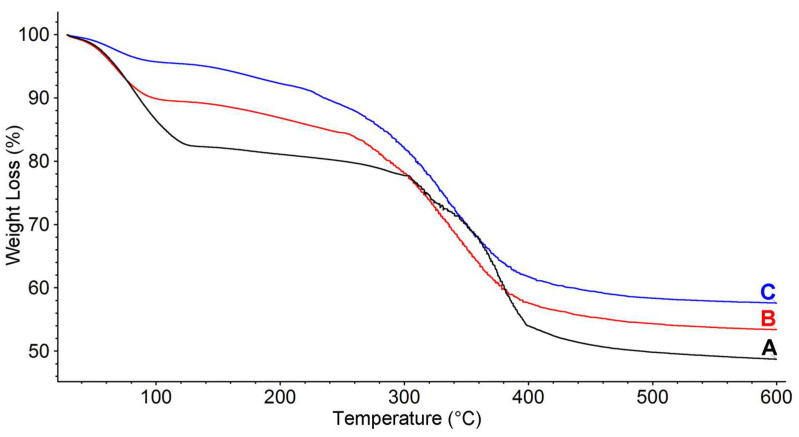
TGA thermograms of SP0 (A, black), SP2 (B, red), and SP3 (C, blue) in a nitrogen atmosphere.

When Si-PEI was analyzed in TGA, the fresh material (SP0) displayed a behavior that closely matches the overlap of the two pure components (Figure 3, black curve), i.e., PEI 5k and silica PQ4. Indeed, besides the initial weight loss attributed to volatiles which was also observed in both the silica and PEI, the sample was stable up to about 300 °C and then underwent a subsequent double weight loss degradation with a pattern strongly reminiscent of the respective pure polymeric component. According to the previous observation that PEI 5k is completely degraded at the end of the heating ramp (Figure S1), TGA provided a silica/PEI ratio of 48.4/51.6, discarding, as previously stated, the volatile fraction. The residue, however, appears dark and thus, applying only the inert condition, some char formation can be inferred.

When analyzing the curves related to the spent Si-PEI solid absorbents SP2 and SP3, the first clear difference with respect to the SP0 sample is the significantly lower volatile fraction, which decreases from SP2 to SP3. Moreover, it can be observed that the two spent absorbents display weight losses starting at a significantly lower temperature, namely around 150 °C, with a degradative pattern that is similar between SP2 and SP3 but is clearly different from SP0 and PEI 5k. All the previously described events compare well with the expected behavior of spent absorbents; indeed, the parasite reactions leading to the lack of performance [26,27,28,37,38] (i.e., non-reversible carbonation reactions and/or interchain crosslinking, which may in turn hamper diffusion) are typically hindering the ability of the PEI fraction to interact not only with CO_2_, but also with volatiles such as humidity, limiting the water uptake ability. It seems clear that the higher the concentration of CO_2_ during the lifetime application, the stronger the impact of such a limitation on the spent product. Furthermore, after the volatile release, the weight loss associated with the PEI-fraction degradation appears at lower temperatures with respect to both the SP0 absorbent and the respective pure PEI 5k component. It is known that the CO_2_ uptake depends on the formation of carbamate ions, which occurs at around 70–80 °C, and can be reversed almost completely above 145 °C [26]. This means that, in the present case, a partial release of some CO_2_ can occur from the adducts formed during the operating life. Discarding the volatile components, when compared to SP0, the solid residue retrieved at the end of the TGA heating ramp is found to be higher in the case of SP2 and SP3 (SP2: 53.1%, SP3: 57.2%), and this effect, once again, can be explained on the basis of the degradative process, implying the intermolecular formation of carbamates, carbamic acids, bicarbonate, zwitterionic structures, and urea moieties, which are not always reversible, thus leading to partial crosslinking and in turn to a higher char fraction.

**Figure 4:**
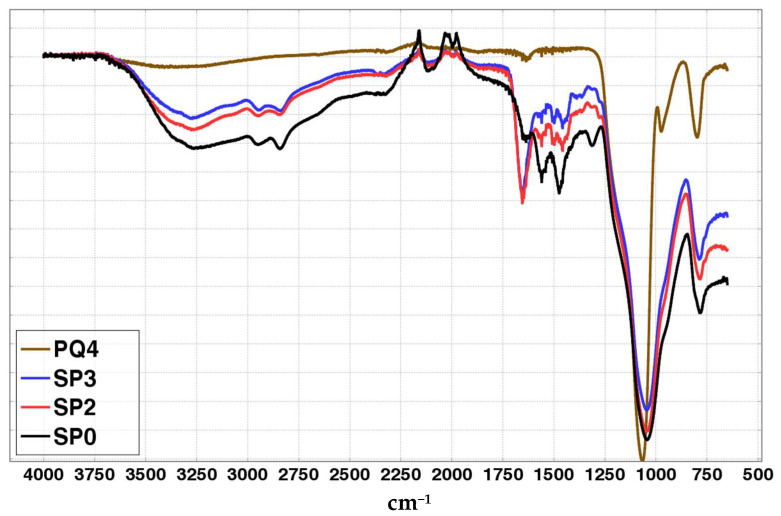
ATR-FTIR plotting of the PQ4, SP0, SP2 and SP3 materials using VMS-DRAW [47].

ATR-FTIR was exploited to gain insights on the chemical composition of such materials. According to Figure 4, all the aforementioned sorbents showed the silica-fingerprinting peaks below 1200 cm^−1^ [39,40,41], consisting of the very intense band at ~1045 cm^−1^ and a weaker one at ~785 cm^−1^ in SPx samples which are lower shifted than the corresponding peaks in pure PQ4 material.

The presence of N-H and aliphatic C-H groups in all SPx materials is confirmed by the observation of the broad band above 3000 cm^−1^ and the peaks at 2800–3000 cm^−1^, respectively [42]. Distinctive features characterizing SP2 and SP3 with respect to SP0 are retrievable by focusing on the 1750–1250 cm^−1^ range spectral shape.

In particular, the presence of urea-type groups in the chains of SP2 and SP3 may be assessed by the observation of an intense peak at 1655 cm^−1^ and a peak at 1560 cm^−1^ (the latter is slightly pronounced/hindered in such samples but clearly observable in the corresponding extracted PEI once lyophilized, see Supplementary Materials), which nicely match the reported DRIFT experiment values (1658 cm^−1^ and 1560 cm^−1^) of a bis-(trimethoxysilylpropyl) urea-grafted pore-expanded MCM-41 mesoporous silica sample [19] and are consistent with other urea-containing systems [43,44]. Similar IR wavenumbers are also observed in prototypical amine-CO_2_ adducts, Ref. [45] thus also suggesting the possible presence of ionic carbamate groups in SP2 and SP3 samples. However, the definite observation of a broad ammonium peak in the 3000–2100 cm^−1^ region [45] has to be proved. On the other hand, in SP0, a side peak at 1640 cm^−1^ seems to be present (plausibly indicating the presence of water [39] as is also observable from the PQ4 sample showing a weak peak at that wavenumber), along with a peak located at 1560 cm^−1^ similar to what was observed in other branched PEIs (PEI 600 and 10,000 MW) and attributed to N-H bending of primary amines [42]. Furthermore, peaks attributable to the -CH_2_- groups [46] at 1474 cm^−1^ and 1313 cm^−1^ are clearly visible in the SP0 spectrum.

### 2.2. Chemical Extraction of PEI from Si-PEI

A protocol to extract the PEI from the SPx samples was developed with the purpose of evaluating the potential of extraction to regenerate silica and valorize PEIs.

It is well known that different parameters contribute to the effectiveness of the extraction procedure, such as the choice of the solvent used. According to the “Technical Information” by BASF [BASF, 08_0806130e-02, September 2010], PEI 5k is soluble in water, methanol (MeOH), and homologue alcohols with up to three carbon atoms, partially soluble in ethyl acetate and insoluble in *n*-hexane, toluene, and xylene, in agreement with the strengths of the classical hydrogen bonds between the respective solvent and PEI.

In this framework, we developed an extraction procedure using MeOH, by combining ultrasound sonication and temperature, as reported in the Section 3. Px identifies the PEI extracted from the corresponding initial sample (SPx) and Sx identifies the silica-remaining counterpart. The extracted PEIs are viscous samples whose color ranges from light yellow to dark brown as shown in Figure S3.

The tested procedure allows the extraction of an amount of PEI about 50%wt of its initial content. In fact, from 3 g of weighted SPx (~40% by weight is the PEI content in each sample as reported in Section 3), extraction yields 50 ± 3%, 50 ± 2%, and 52 ± 4% for P0, P2, and P3, respectively.

Microscopical observations of the SPx and Sx samples (Figure S4) showed an increase in transparency within each of the given samples. These materials have been used to perform all of the characterization unless otherwise stated.

The performed PEI extraction is also deducible from the ATR-FTIR spectra of Sx, which shows either a decrease or a disappearance of PEI peaks at around 3280 cm^−1^ and in the 1700–1250 cm^−1^ range.

Peaks of silica (see previous paragraph) were not detected in Px samples. This finding was confirmed by applying the same extraction protocol to PQ4: the extraction yield was indeed less than 1% by weight.

The closer similarity between PQ4 and the Sx samples rather than the SPx samples was deducible by the less-shifted values of the aforementioned Si-O-Si peaks.

#### 2.2.1. TGA

**Figure 5:**
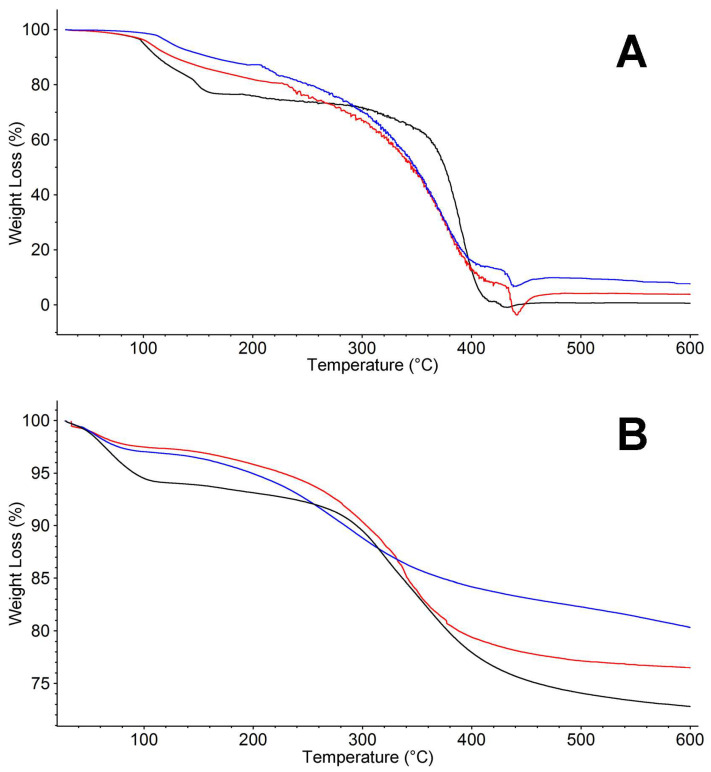
TGA thermograms of (**A**) P0 (—), P2 (—), and P3 (—) in nitrogen atmosphere and (**B**) S0 (—), S2 (—), and S3 (—).

As shown in Figure 5A, the TGA of P0 extract (black line) is similar to the pristine polymer PEI 5k (see Figure S1), and is in turn similar to SP0, showing a preliminary volatile release and then, at about 300 °C, the substantial chain degradation; it is, however, worth pointing out that, while there is still a double-loss event in the 300–400 °C temperature range, the entity of the first drop is strongly limited with respect to the previous cases. This finding suggests that the extracted polymer fraction is somehow different from the bulk polymer, probably owing to some fractionation. The two P2 and P3 extracts (Figure 5A, red and blue lines) are once again very similar one to another, quite reminiscent of the degradative behavior of SP2 and SP3 and different from P0, but also from SP0 and commercial PEI 5k. Such an observation supports the idea of extracting a partially degraded polymeric fraction from the spent absorbent. Hence, while forming parasite crosslinks, it seems that a fraction of the polymeric mass is still not part of a crosslinked network and can be released in solvent.

The weight loss at low temperatures of S0, S2, and S3 is significantly lower than in SP0, SP2, and SP3.

The quantities of PEIs remaining in Sx was calculated from the weight loss in the temperature range of PEI thermal decomposition. The non-extracted PEIs totaled 29%, 25%, and 25% for S0, S2, and S3, respectively. These values are in line with 50% of the polymer mass being removed with extraction.

As to char fraction, simulations of pyrolytic conditions by TGA experiments (Figure S5) confirmed that the combination of chemical extraction followed by thermal treatment for each of the SPx samples resulted in up to a ~2% reduction of char content with respect to the only thermal process as shown in Table 1

#### 2.2.2. Rheology

Physical behavior similarities between P2 and P3 with respect to P0 have also been pointed out by investigating the viscosity of such materials at 23 °C. For this purpose, extracted PEIs were lyophilized to optimally minimize the variability in viscosity due to the presence of the solvent.

**Figure 6:**
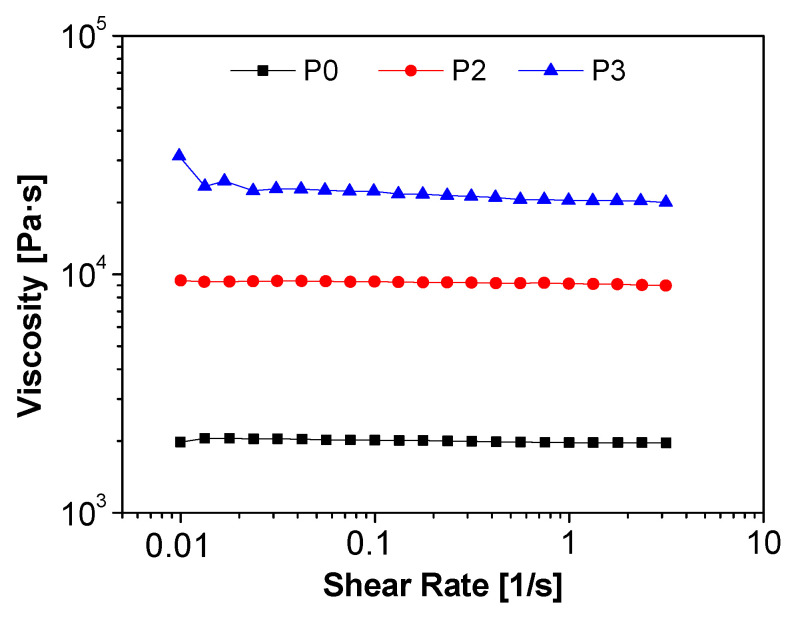
Viscosity curves of the lyophilized extracted PEI: P0 (black squares), P2 (red circles) and P3 (blue triangles).

Figure 6 highlights an increase in viscosity moving from sample P0 to P2 and P3, in relation with the presence and quantity of adsorbed CO_2_. This behavior was reported for different systems, such as amine solutions [48,49], ionic liquids [50,51], and PEI/PEG blends [52] where the increase in viscosity is due to the formation of carbamate ion pairs, which leads to cross-linking between the polymer chains.

#### 2.2.3. ATR-FTIR

Looking at the molecular level, the ATR-FTIR spectra of P0, P2 and P3 (Figure S6) show similar shapes with respect to the corresponding SPx, with a better definition of peaks, even if slight wavenumber shifts or intensity differences are observable. Overall, the IR molecular fingerprinting was not markedly affected by the extraction, confirming the close similarity between P2 and P3, and their differences with P0, especially the presence of the distinctive degradation peak at 1655 cm^−1^. As expected, the strong silica-related peak below 1200 cm^−1^ is missing. Interestingly, the broad band of N-H stretching modes narrowed in shape, allowing a better evaluation of the N-H group type present in the samples. In fact, the presence of primary [46,53] and secondary N-H_2_/N-H groups [46] in all Px samples is consistent with the shape of the band with maximum transmittance at around 3280 cm^−1^.

#### 2.2.4. ^13^C-AND ^1^H-NMR

Further fingerprints of the chemical structure of the extracted PEI are provided by analysis of the ^13^C NMR spectra of Px in comparison to many other branched PEIs reported in the literature [54,55,56].

**Figure 7:**
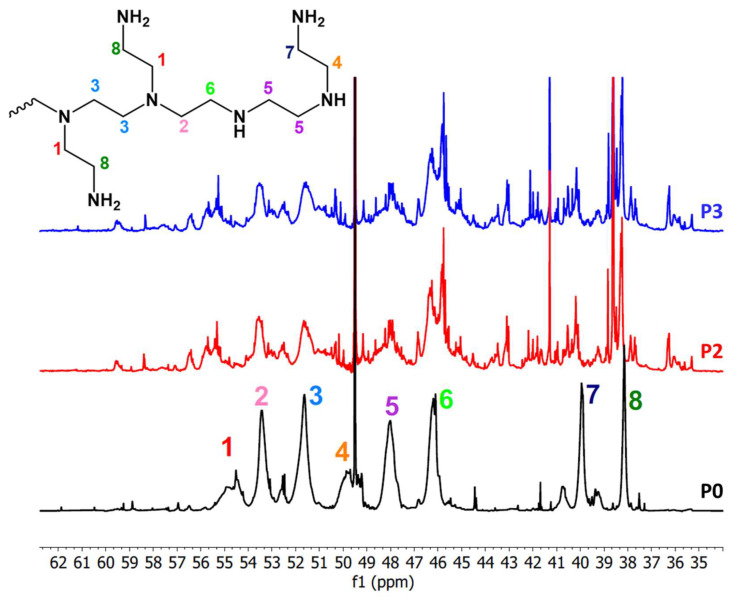
^13^C NMR spectrum (zoom between 35 and 62 ppm) of samples P0 (black), P2 (red), and P3 (blue). The peak numbers indicate the carbon attribution given in the inset.

The chemical shifts of the aliphatic region are characterized by the presence of alkyl groups of PEIs adjacent to the primary amines (38–40 ppm), secondary amines (45–53 ppm) and tertiary amines (53–57 ppm) [54,55,56] (Figure 7).

In the same aliphatic region, the spectra of P2 and P3 are very different (Figure 7). After the CO_2_ adsorption, all the peaks that were neatly in the aliphatic region of P0 are still present, but they lose resolution and decrease in intensity, resulting in the splitting of many other smaller peaks. As it was also observed for the ^1^H spectrum (Figure S7), the complex pattern of the peaks may arise from the binding of CO_2_ to the polymer chains, producing several new functional groups [26,27,28,38]. The peaks at 51 and 53 ppm, deriving from carbon atoms attached to tertiary amines, are less affected by CO_2_ adsorption, probably because tertiary amines are not involved in the reaction with CO_2_ [28].

Moving to larger ^13^C NMR chemical shift values (Figure S8), the peak of P0 at 165.0–165.2 ppm is attributable to CO_2_ adsorption from ambient air during storage or the extraction period. This region of the spectrum (between 157 and 178 ppm) becomes more complicated for samples P2 and P3, with the appearance of many other peaks due to the oxidation process and the splitting of existing ones [28,37,57].

In general, the region 148–180 ppm can be attributed to quaternary carbons and to CH=N- groups. The peaks of the C=O deriving from the CO_2_ bonded to the polymer chains were previously reported to be in two main areas: (i) around 159.5–161.0 ppm, attributed to disubstituted and trisubstituted open-chain ureas and N,N′-disubstituted cyclic ureas, and (ii) around 160–163 ppm, attributed to N-substituted cyclic ureas [28].

Similar conclusions can be deduced from the analysis of the ^1^H NMR spectra of P0, P2, and P3 (Figure S7). The peaks between 2.5 and 3.0 ppm in the fresh sample can be attributed to the protons in methylene groups all having a similar environment. The peak at 3.33 ppm is some residual methanol, not completely evaporated after the extraction from silica. New peaks appear in the area 3.2–3.7 ppm, as well as in the ranges 0.7–2.4 ppm and 7.5–8.5 ppm in the spectra of samples P2 and P3, corresponding to the formation of new functional groups after the binding of CO_2_ to the polymer chains, which produces carbamates, carbamic acids, bicarbonate, zwitterionic structures, and urea linkages [26,28,37,38].

#### 2.2.5. Py-GC-MS

Analytical pyrolysis of PEIs has recently been reported in the literature [58,59].

In agreement with these studies, the relevant pyrolysis products of the PEI are alkylated pyrazines, while pyridines, pyrroles, and imidazoles were minor aromatic components (an exemplar pyrogram of fresh PEI 5k is reported in the supplementary information, Figure S9). Besides aromatic amines, the pyrolysate featured aliphatic cyclic (piperazine moiety) and linear polyethyleneimine oligomers.

**Figure 8:**
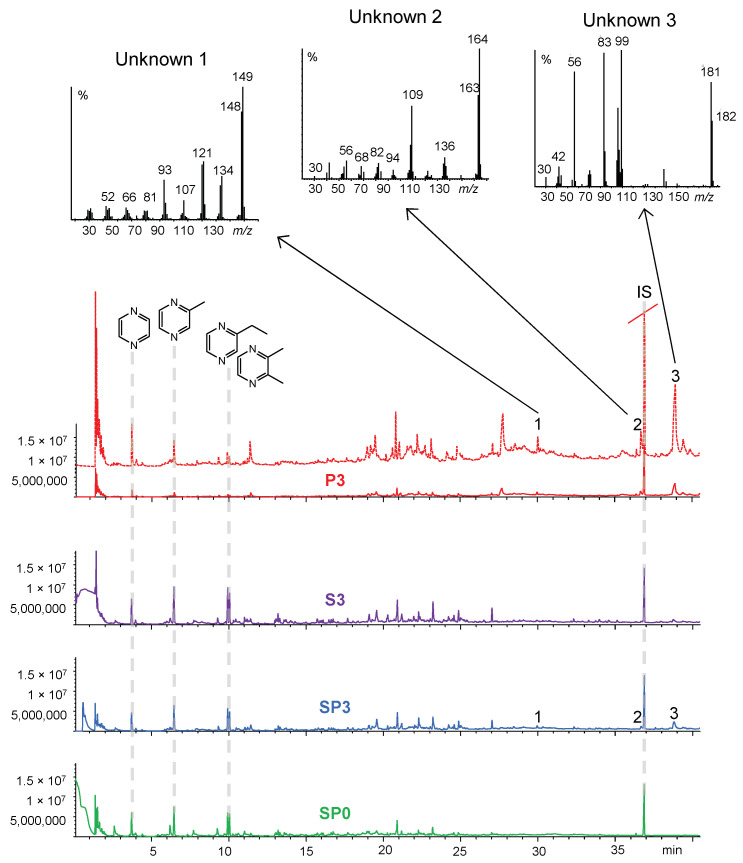
MS-pyrograms from Py-GC-MS at 600 °C of (from bottom to top) SP0 (green), SP3 (light blue), S3 (purple), P (red). The magnified pyrogram of P3 is also shown (red dotted line) with the mass spectra of GC peaks #1, #2, and #3, indicative of degradation. IS stands for internal standard.

Pyrazines became predominant pyrolysis products in the pyrolysates of pre- and post-extracted Si-PEI due to the action of mesoporous silica that promoted alkylation and aromatization reactions [58]. Examples of pyrograms are shown in Figure 8. Pyrazines were important components of the pyrolyzates of the extracted PEI, but alkylation was less pronounced and thermal degradation products at higher retention times were relatively abundant (Figure 8). In addition to the typical pyrolysis products of the fresh PEI, the pyrograms of spent samples presented more intense (#1 and #2 in Figure 8) or novel peaks (e.g., #3). The molecular identity of these additional peaks could not be assigned, but they could be considered proxies of the degradation processes.

Alkylpyrazines are chemicals of potential applicative interest for the purpose of valorizing spent Si-PEI adsorbents by pyrolysis. In this framework, for instance, 2,3-dimethylpyrazine and methylpyrazine account for two of the most processed flavoring alkylpyrazines [60].

The yields of pyrazines evolved from the pyrolysis of the PEI were estimated by single point calibration with an internal standard. The yields of silica-PEI were 12.1%, 6.3%, and 6.8% for SP0, SP2, and SP3, respectively, with an apparent decrease due to degradation.

The pyrazine yields for the extracted PEIs were significantly lower (3.0%, 2.8%, and 2.7% for P0, P2, and P3, respectively). This result confirmed the effect of mesoporous silica to promote the formation of pyrazines. The lower pyrazine yields from the extracted PEIs are balanced by a higher production from the PEI entrapped in the silica network that could not be extracted. The yields from the spent sorbent increased from 6–7% to 15% (S2) and 11% (S3). In general, the yields were not high in absolute terms, but fully comparable to the yields of chemicals obtainable form the pyrolysis of complex polymeric materials as cellulose or biomass [36].

## 3. Materials and Methods

**Samples**. The samples under investigation consist of Si-PEI sorbents, produced from a PQ4 silica matrix of commercial origin impregnated with PEI 5k (5000 Da molecular weight, BASF) of ~40% by weight (see below for the exact mass fractions of each sample) [26]. The other three samples were obtained after the use of such Si-PEI for CO_2_ capture in a lab-scale twin bubbling fluidized-bed system [61] using different amounts of carbon dioxide.

Overall, the samples (SPx) are so composed:

- SP0: fresh Si-PEI, 42.99 ± 0.06 wt% loading dried base.
- SP2: spent Si-PEI 42.5 ± 0.2 wt% loading 2%wt CO_2_.
- SP3: spent Si-PEI 42.8 ± 0.2 wt% loading 3%wt CO_2_.

PQ4 and PEI 5k were also characterized to act as reference materials for the investigation of Si-PEI samples (SPx), their extracted polymers (Px) and the remaining silica (Sx).

**PEI extraction.** The extraction procedure was performed by adding, in 100 mL round-bottomed flasks, 3 g (exactly weighted) of the different SPx samples (and PQ4 as a procedural blank) and 60 mL of MeOH. All samples were sonicated for 1 h starting from 40 °C up to 50/55 °C at the end of the process. For each sample, the supernatant was separated from the powder deposited on the bottom of the flask by filtration using a “Whatman 5 qualitative 110 mm” filter at room temperature. Successively, the wet powder and the flask were washed by adding 2 × 10 mL of MeOH, whose fractions were also put through the filter. The collected liquid fractions were centrifugated at 4400 RPM for 10 min at about 8 °C and then the liquid was rotary evaporated and dried. The obtained viscous liquids, consisting of extracted PEIs, were named Px, while the remaining (from starting) rotary-evaporated solid counterpart was named Sx.

**TGA.** The samples’ thermal stability was evaluated via TGA analysis using a thermogravimetric apparatus (TA Instruments Q600, New Castle, DE, USA), under a nitrogen and/or air atmosphere condition (flow rate 20 mL/min), by heating the sample (10 mg) at a 10 °C/min heating rate, from 25 °C to 600 °C or 850 °C.

The TGAs related to the spent Si-PEI solid absorbents (SP0, SP2, and SP3) were carried out under the nitrogen condition by heating the sample at a 10 °C/min heating rate, from 25 °C to 600 °C, followed by a 2 h isotherm in the nitrogen condition, a heating ramp at a 10 °C/min heating rate from 600 °C to 850 °C in air condition, and an isotherm 1 h step, again in air atmosphere.

The TGAs of the extracts P0, P2, and P3 and the TGAs of the S0, S2, and S3 were carried out under a nitrogen condition by heating the sample at a 10 °C/min heating rate, from 25 °C to 600 °C.

**Optical Microscope.** SPx and Sx samples were deposited on microscopy glass and the optical microscope images were recorded using a Nikon (Minato, Japan) 13 ECLIPSE Ti2 Inverted Research Microscope with a 10× magnifier.

**Viscosity.** The viscosities of the extracted PEIs were investigated using an Anton Paar (Graz, Austria) MCR102 rheometer. A cone/plate (CP 25-1, diameter = 25 mm, α = 1°) measuring geometry was used, with a fixed gap of 0.049 mm. Viscosity curves were recorded at a fixed temperature of 23 °C, controlled by an integrated Peltier system, and a shear rate in the range 0.01–3.00 s^−1^.

**ATR-FTIR spectroscopy**. ATR-FTIR spectra (% transmittance) have been collected in the 4000–650 cm^−1^ range using an Agilent (Santa Clara, CA, USA) Cary 630 FTIR spectrometer (64 scans, 4 cm^−1^ resolution, ATR diamond crystal). Spectra have been overlapped by vertically shifting them to have the same starting point at 4000 cm^−1^.

**NMR spectroscopy**. NMR spectra were recorded with a Brucker Ascend 600 spectrometer at 600 MHz (^1^H NMR) and at 150 MHz (^13^C NMR). The probe is a cryoprobe PRODIGY broadband cooled with liquid nitrogen. All extracted PEI samples were dissolved in D_2_O (4.79 ppm).

**Pyrolysis.** Py-GC-MS of SPx, Sx and Px was performed with a multi-shot pyrolizer (EGA/PY-3030D Frontier Lab, Fukushima, Japan) connected to a gas chromatograph-mass spectrometer (7890B-5977B Agilent Technologies). An aliquot of samples (about 0.5 mg of SPx and about 1.5 mg of Sx both exactly weighed; 5 μL of Px MeOH solution 0.5 mg mL^−1^) was introduced into the pyrolysis cup. The sample was spiked with the internal standard solution (2.5 μL of 125 µg mL^−1^ methyl palmitate from Sigma Aldrich in MeOH, St. Louis, MO, USA). Samples were pyrolyzed at 600 °C under 1:50 split conditions. Pyrolysis products were separated with a HP-5MS GC-MS column and mass spectra were recorded under 70 eV electron ionization in the *m*/*z* 30–600 interval under the same conditions described by Coralli et al., 2023 [58]. The yields of alkylpyrazines were estimated from single-point calibration using methyl palmitate as the internal standard. A calibration solution of 2-methylpyrazine, 2,3-dimethylpyrazine, 2,5-dimethylpyrazine, 2-ethylpyrazine, 2-propylpyrazine, 2-ethyl-3-methylpyrazine (Sigma-Aldrich), exactly measured at about 0.3 mg mL^−1^ each, was analyzed in triplicate and the response factor relative to methyl palmitate was determined to calculate the quantity of pyrazines evolved from the pyrolysis of a known sample amount. Yield values were reported relative to the quantity of pyrolyzed PEIs. Relative standard deviation was below 23%, which is satisfactory for Py-GC-MS.

## 4. Conclusions

In the present paper, Si-PEI was investigated as a valuable solid sorbent technology for CO_2_ adsorption. The study aimed at providing insight into the potential valorization of the spent material, through the regeneration of the silica and the production of exploitable chemicals. Two possible routes (solvent extraction and pyrolysis) and their combination were explored.

The proposed solvent extraction with methanol is a simple method able to recover a 50%wt of polymer which enabled a detailed characterization of the degraded PEI by different techniques.

For a quantitative recovery of PEIs and efficient regeneration of silica, different factors could be worth consideration, such as higher temperatures, sequential extractions, and other solvents.

The chemical extraction allowed us to envisage two fractions of PEI in the solid sorbent: a solvent-transferable portion (loosely bound to the solid matrix) and a refractory portion (cross-linked, occluded, or tightly bound). The proportion of these two fractions was not severely altered by oxidation.

Notably, pyrolysis of the PEI produced chemicals of potential applicative interest, such as alkylpyrazines. Qualitatively, the same chemicals were generated from pyrolysis of Si-PEIs, extracted PEIs, and the refractory polymer in silica post-extraction, regardless of the oxidation degree of the materials.

The degradation of sorbents, due to repeated CO_2_ adsorption/regeneration cycles, did not reduce the yield of pyrazines. However, the presence of mesoporous silica is fundamental to favor pyrolysis yields.

Pyrolysis of the extracted sorbents (Sx) resulted in a lower production of coke in comparison to the untreated samples (SPx). Nevertheless, the coke residue must be removed through combustion to fully regenerate silica support.

Overall, the results provided the basis for future investigations on the potential routes to valorize spent-solid adsorbents in CCS technology.

## Figures and Tables

**Table 1:** Char formation in non-treated (SPx) and in chemically treated (Sx) samples.

Sample	Char (%)
SP0	3.2
S0	2.6
SP2	5.1
S2	2.8
SP3	5.3
S3	4.0

## Supplementary Materials

The following supporting information can be downloaded at: https://www.mdpi.com/article/10.3390/ijms241914415/s1.
