# Supplementary material for: Valorization Strategies in CO2 Capture: A New Life for Exhausted Silica-Polyethylenimine
Source: Int J Mol Sci. 2023 Sep 22;24(19):14415. doi: 10.3390/ijms241914415 (PMC10572583; doi:10.3390/ijms241914415)
Supplement: Supplementary file 1 [file ijms-24-14415-s001.zip › ijms-2565880-supplementary.pdf]

Supporting Information

Valorization Strategies in CO_2 capture: A New Life for Exhausted Silica-Polyethylenimine

Irene Coralli,[1] Demetra Giuri,[1,*]Lorenzo Spada,[1,*]Jacopo Ortolani,[2,] Laura Mazzocchetti,[2] Claudia Tomasini,[1] Lee A. Stevens,[3] Colin E. Snape,[3] Daniele Fabbri.[1]

1 Department of Chemistry "Giacomo Ciamician", University of Bologna, Technopole of Rimini, via Dario Campana 71, 47922 Rimini, Italy.
2 Department of Industrial Chemistry "Toso Montanari", University of Bologna, Viale Risorgimento 4, 40136 Bologna, Italy
3 University of Nottingham, Faculty of Engineering, The Energy Technologies Building, Nottingham NG7 2TU, United Kingdom.

Table of Contents

**Figure S1.** TGA thermogram of PEI 5k in nitrogen atmosphere	S2
**Figure S2.** TGA thermogram of Silica PQ4	S2
**Figure S3.** Example of the extracted PEI	S3
**Figure S4.** Optical microscope images of Si-PEI	S3
**Figure S5.** TGA thermograms of SPx thermo-degraded in nitrogen atmosphere	S4
**Figure S6.** ATR-FTIR of the extracted PEI	S5-6
**Figure S7**. ^{1}H NMR spectrum of the extracted PEI	S7
**Figure S8**. ^{13}C NMR spectrum of the extracted PEI	S7
**Figure S9.** MS-pyrograms from Py-GC-MS at 600 °C of PEI 5k	S8

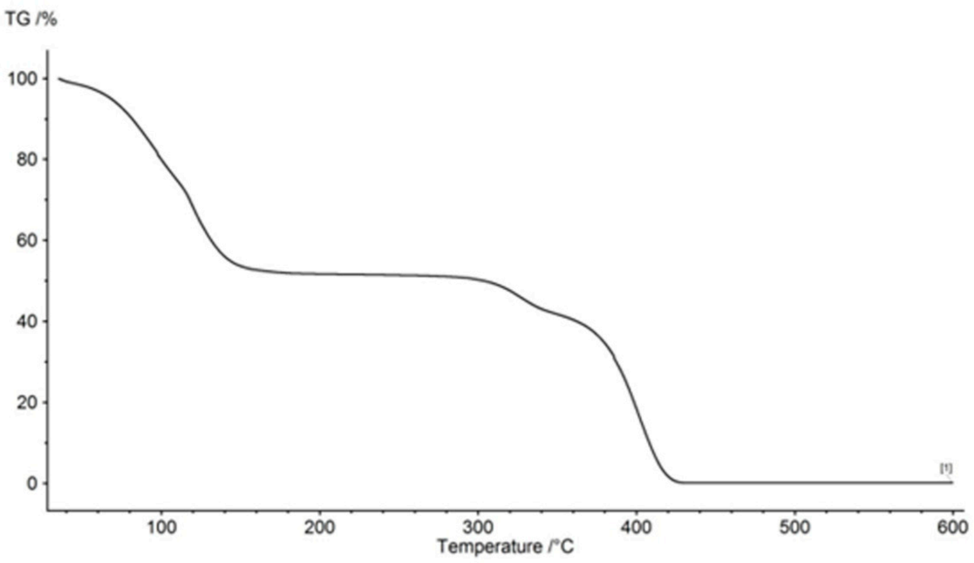

Figure S1. TGA thermogram of PEI 5k in nitrogen atmosphere.

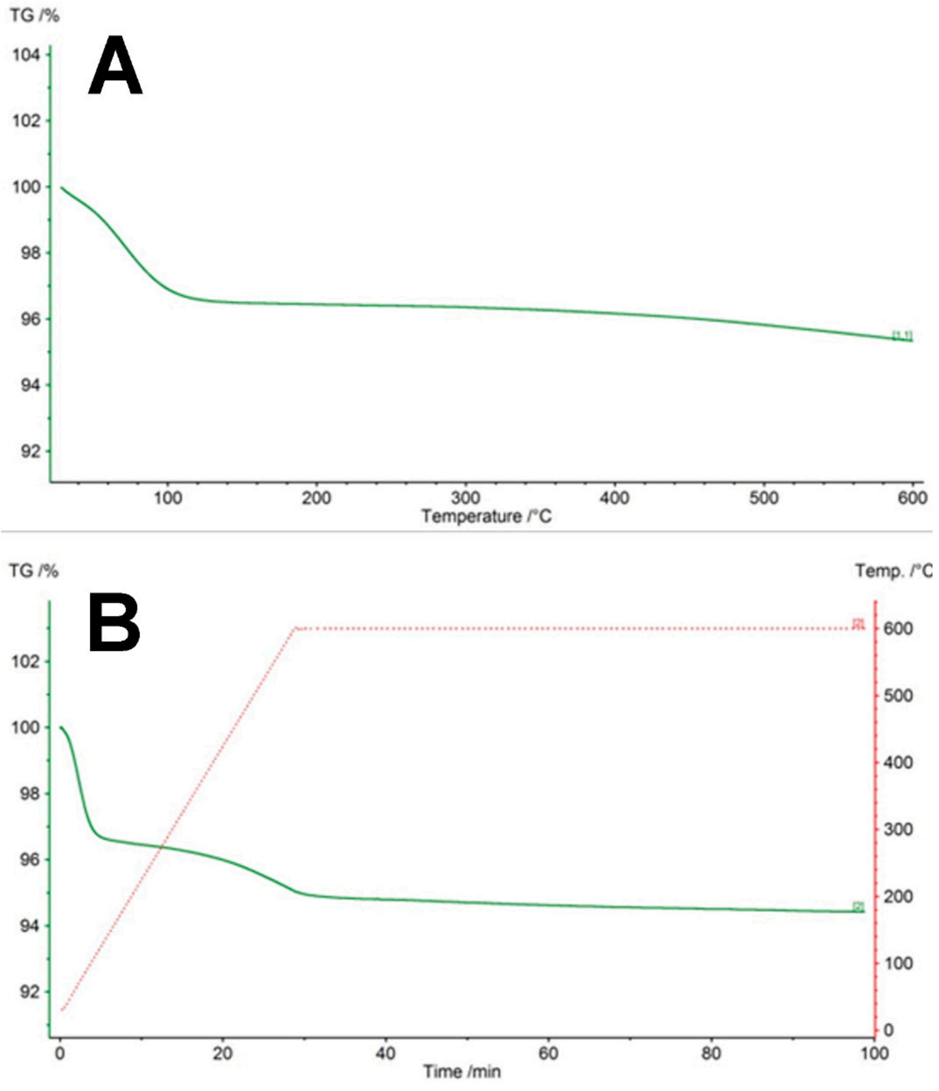

Figure S2. TGA thermogram of Silica PQ4: A) heating ramp in nitrogen atmosphere; B) the whole analysis comprising a first heating ramp and the subsequent isotherm step in air. The latter is in time scale, and in red dotted line the applied temperature ramp is described.

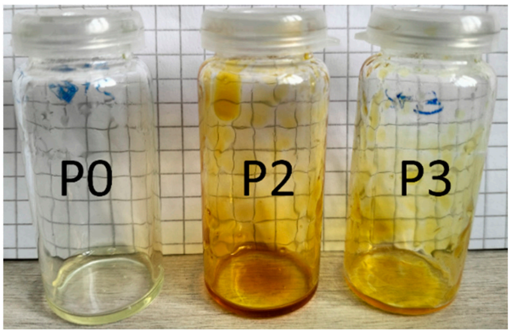

Figure S3. Example of the extracted polymers: P0, P2 and P3.

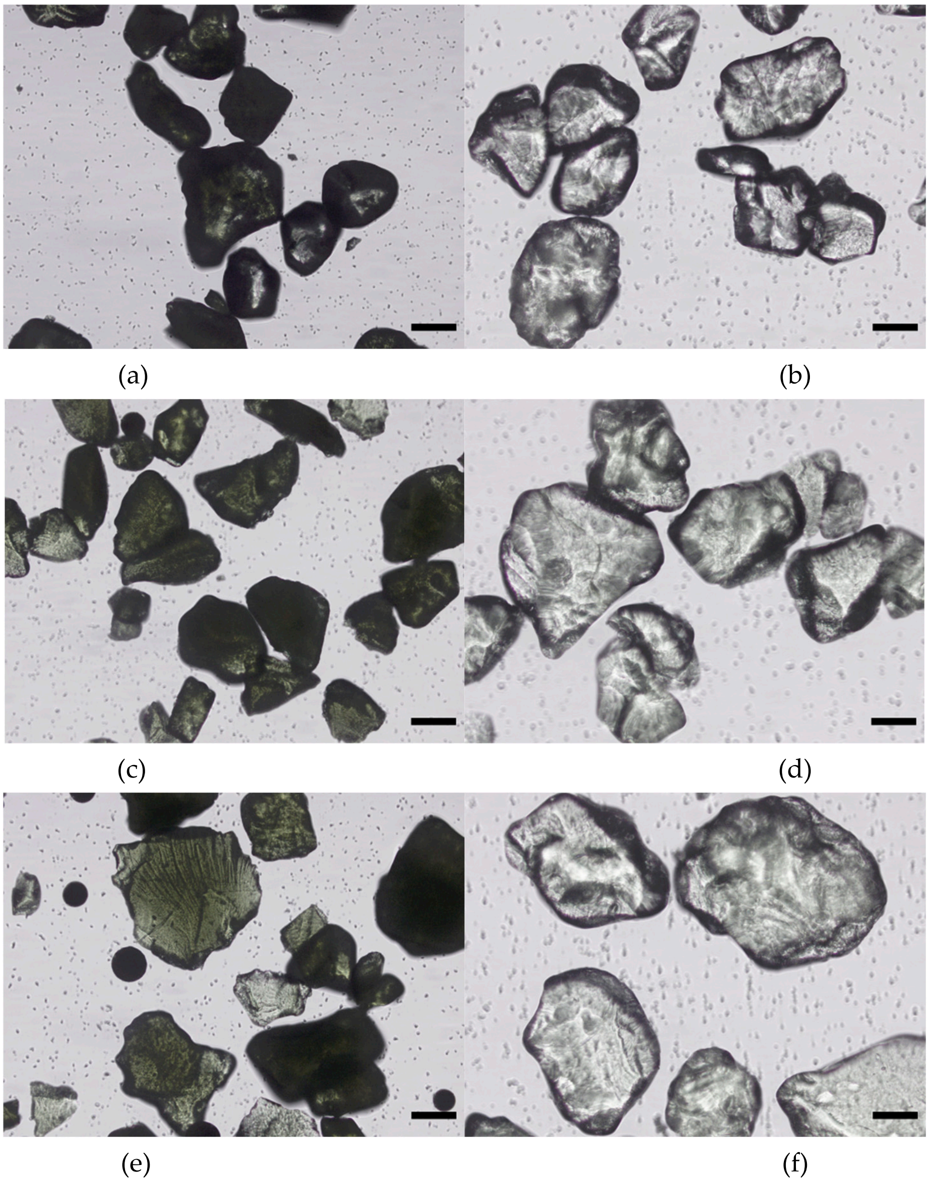

Figure S4. Optical microscope images of pre- and post-extraction grains of Si-PEI samples, left and right column, respectively. The pictures (a) and (b), (c) and (d), (e) and (f) correspond to the microscopically magnified SP0 and S0, SP2 and S2, SP3 and S3 samples, respectively. Scalebar: 100 μm.

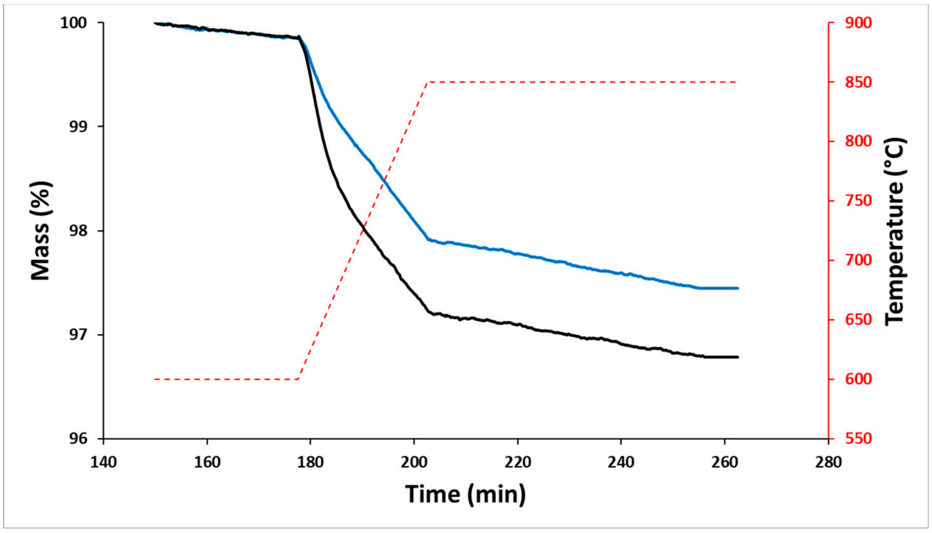

SP0

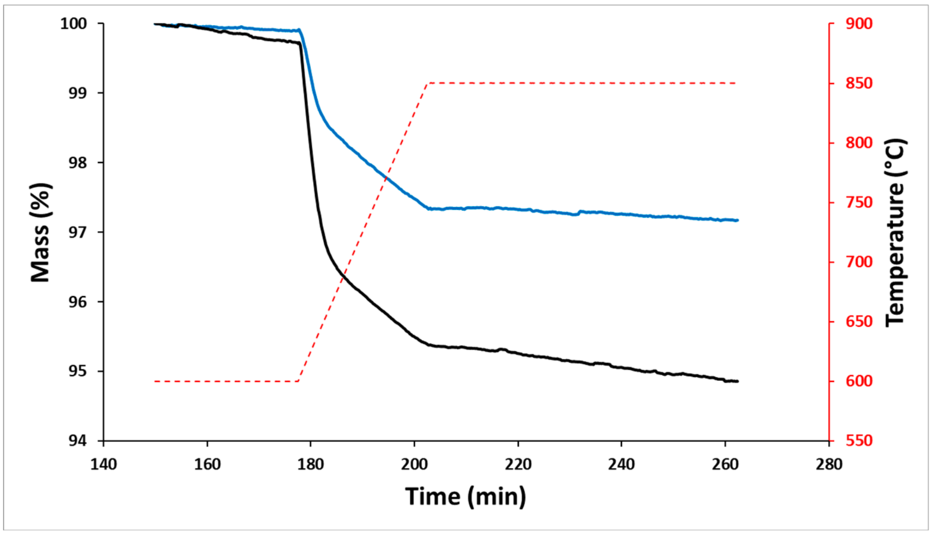

SP2

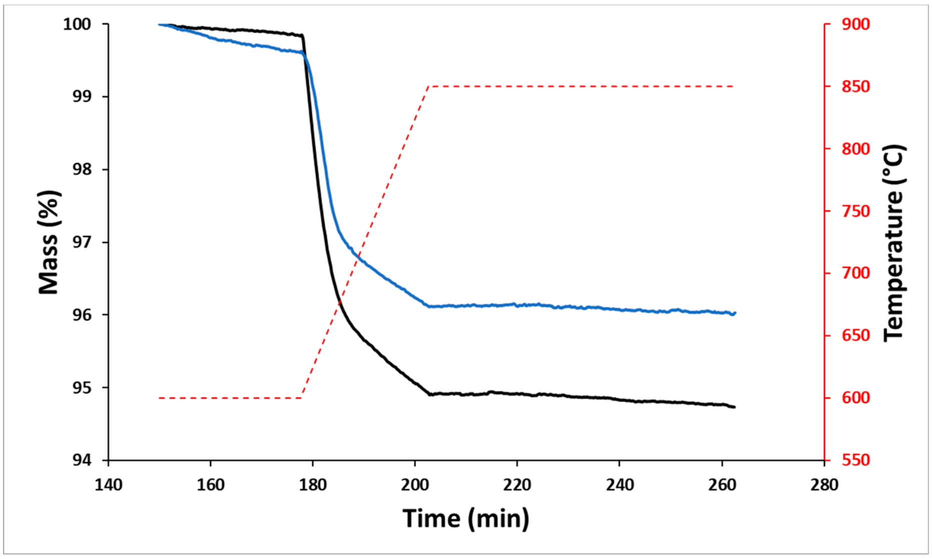

SP3

Figure S5. TGA thermograms of SPx thermo-degraded in nitrogen atmosphere in order to simulate a pyrolysis cycle.

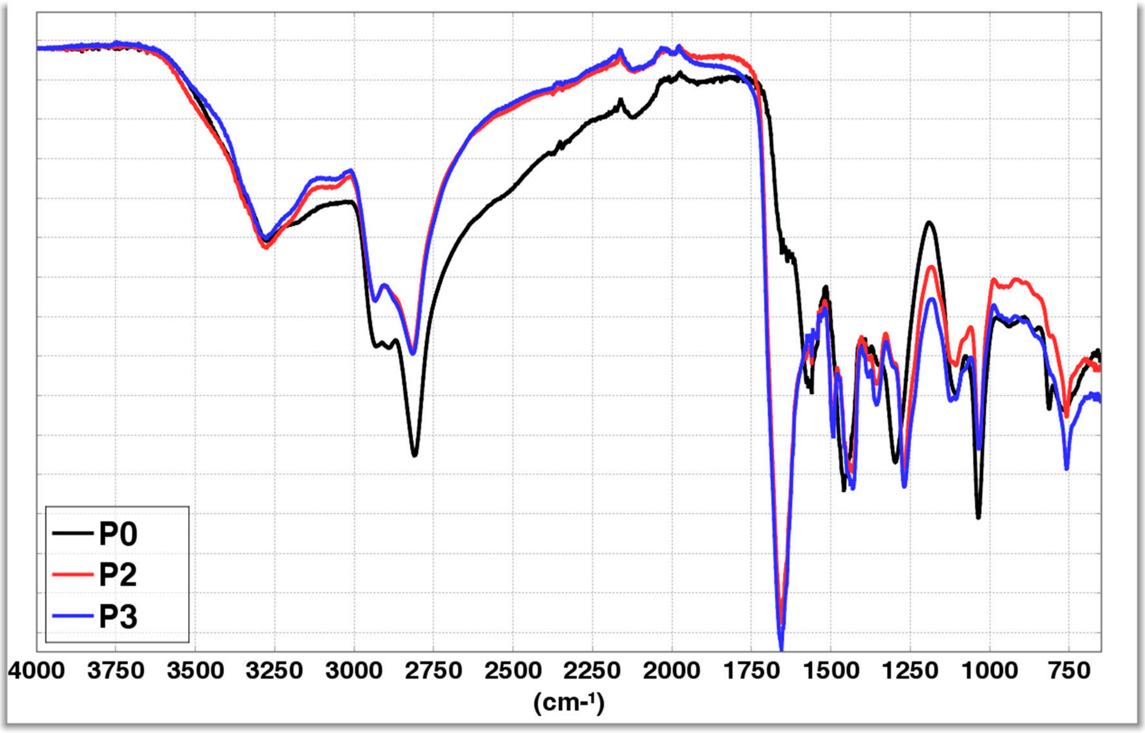

P0
P2
P3
4000
3750
3500
3250
3000
2750
2500
2250
2000
1750
1500
1250
1000
750
(cm-1)

(a)

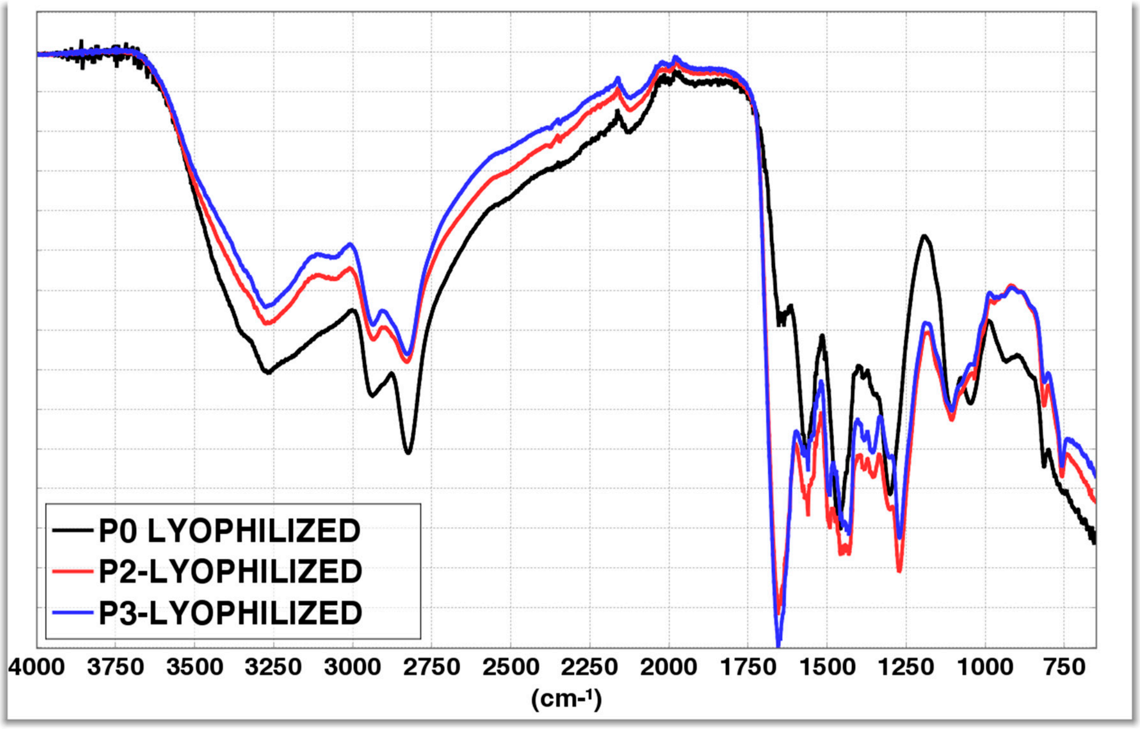

P0 LYOPHILIZED
P2-LYOPHILIZED
P3-LYOPHILIZED
4000
3750
3500
3250
3000
2750
2500
2250
2000
1750
1500
1250
1000
750
(cm-1)

(b)

(c) 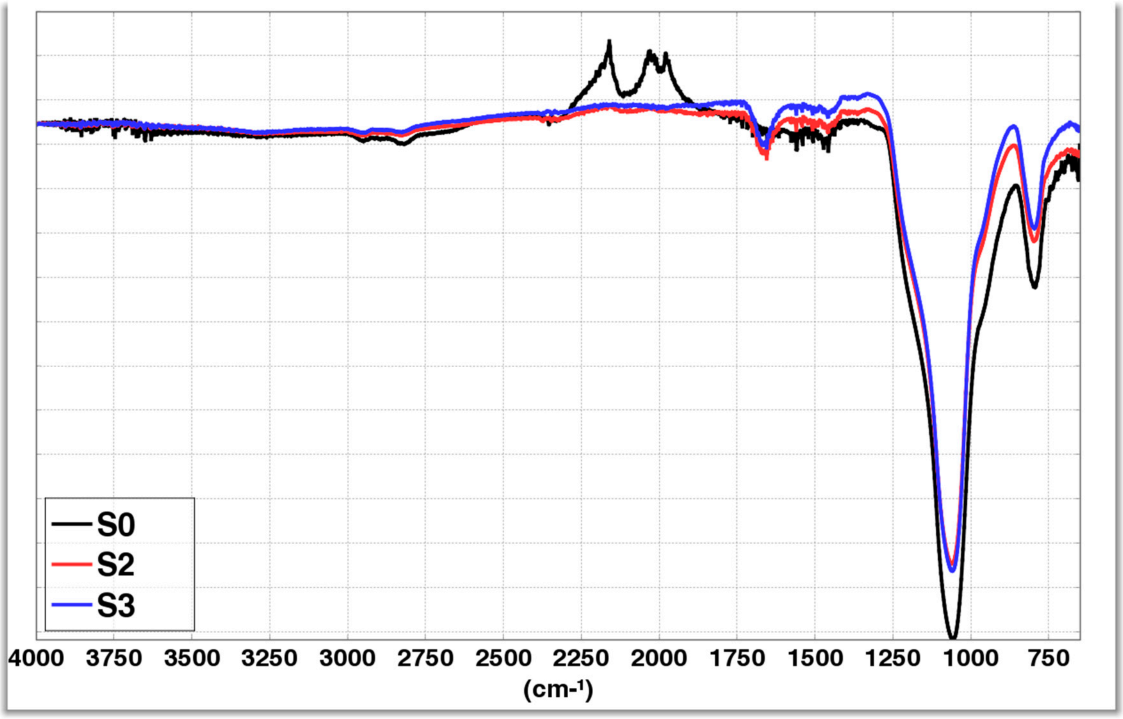

Figure S6. ATR-FTIR of the extracted PEI (Px, a), lyophilized extracted PEI (lyophilized Px, b) and silica-remaining sample after PEI extraction (Sx, c). VMS-DRAW [1] has been used to plot the corresponding spectra.

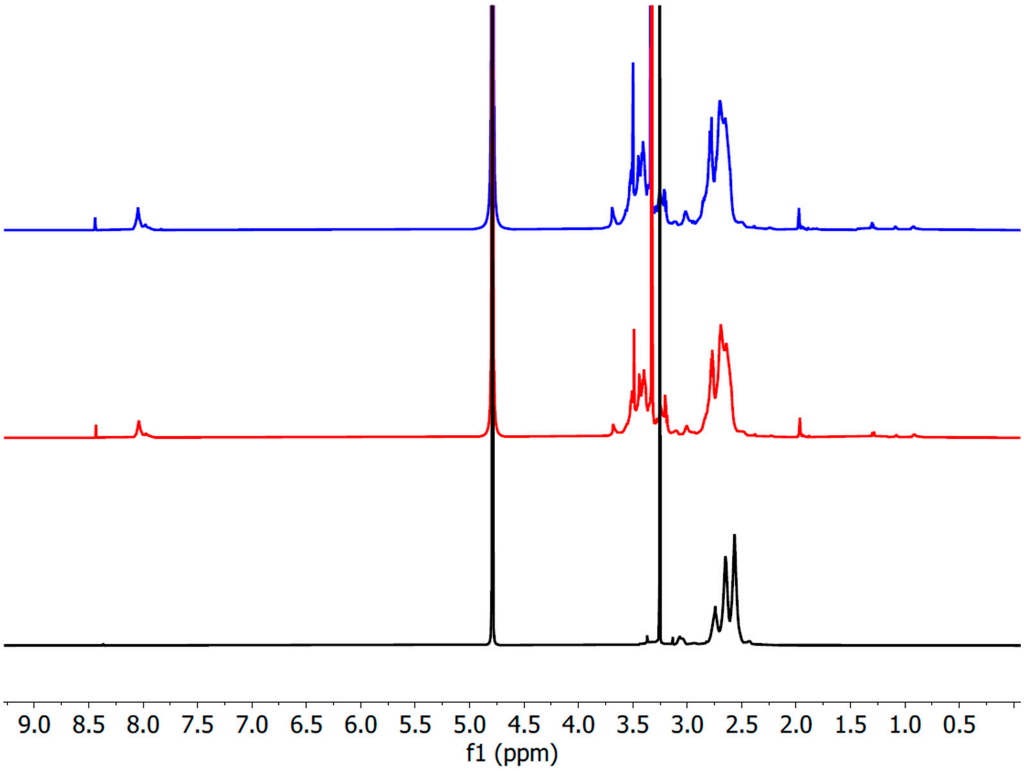

Figure S7. ^{1}H NMR spectrum of extracted PEI samples: P0 (black), P2 (red) and P3 (blue).

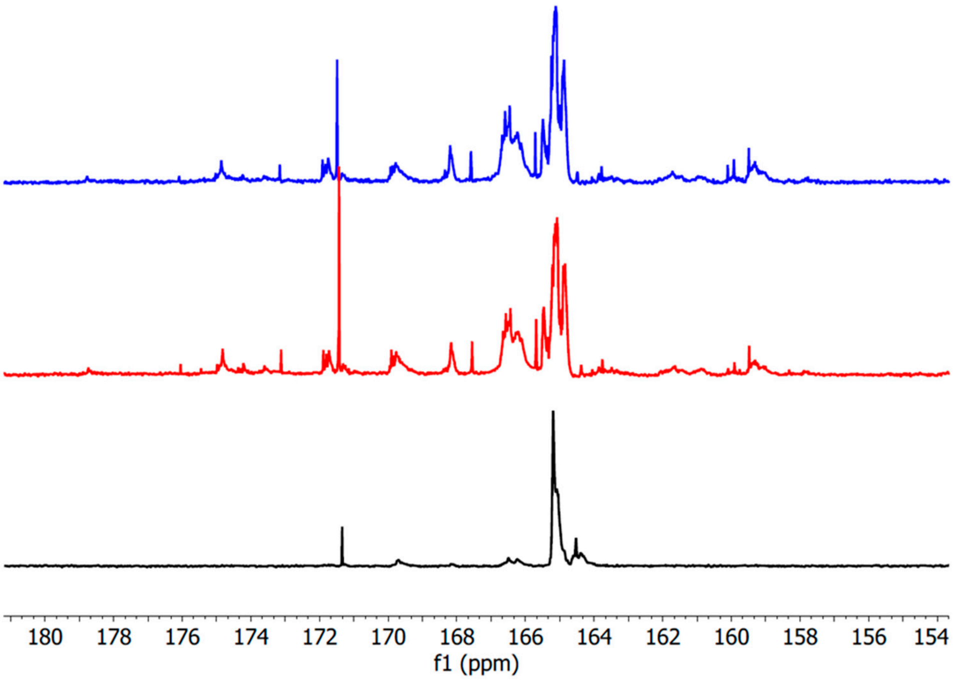

Figure S8. ^{13}C NMR spectrum (zoom between 155 and 180 ppm) of extracted PEI samples: P0 (black), P2 (red) and P3 (blue).

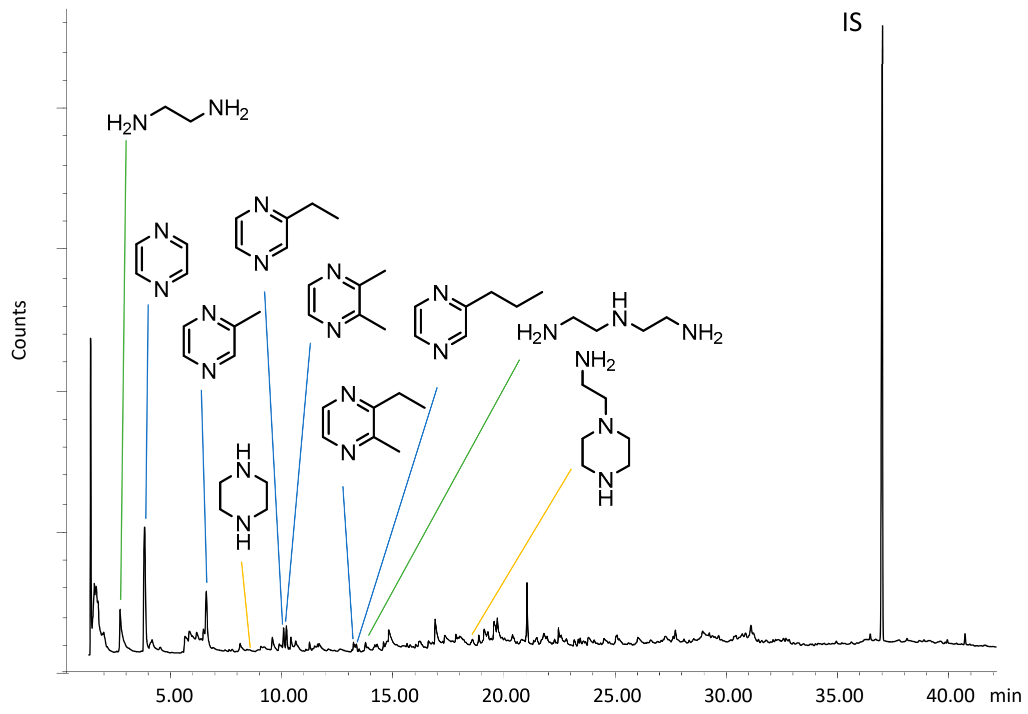

Figure S9. MS-pyrograms from Py-GC-MS at 600 °C of PEI 5k. IS stands for internal standard.

Reference:

1. Licari, D.; Baiardi, A.; Biczysko, M.; Egidi, F.; Latouche, C.; Barone, V. Implementation of a Graphical User Interface for the Virtual Multifrequency Spectrometer: The VMS-Draw Tool. *J Comput Chem* **2015**, *36*, 321–334, doi:https://doi.org/10.1002/jcc.23785.
